# Impact of respiratory syncytial virus in an open bay neonatal intensive care unit

**DOI:** 10.1017/ash.2023.360

**Published:** 2023-09-29

**Authors:** Minisha Morris, Annette Vasandani, Yvonne Estrada, Malik Merchant, Annie Garcia, John Duran, Melissa Gallant, Adonica Benesh

## Abstract

**Background:** Respiratory syncytial virus, RSV, is a respiratory virus that causes cold-like symptoms in adults. In infants and young children, RSV can cause severe illnesses such as bronchiolitis or pneumonia. We describe a successful response to a laboratory-confirmed RSV outbreak in a 21-bed open-pod neonatal intensive care unit (NICU) at a level 2 trauma hospital. **Methods:** After 2 of the 3 initial neonates were diagnosed with hospital-onset RSV, an outbreak investigation began on November 16, 2022. Following the results, testing was expanded to all neonates in the NICU. The clinical case was defined as a hospitalized neonate with laboratory confirmation of RSV by RSV antigen screen or polymerase chain reaction (PCR) detection on the Biofire respiratory panel. Outbreak resolution was determined by utilizing a viral test for the remaining positive neonates after the 2-week incubation period from the last identified positive neonate. **Results:** On day 1 of the investigation, 6 of 18 neonates were identified as positive for RSV. The initial 12 negative neonates received a prophylactic dose of palivizumab. Due to the increase in positive neonates, enhanced infection prevention and control measures were immediately implemented. These measures included the immediate closure of the NICU for new transfers, placing all positive neonates in a single-bay cohort in the NICU, implementing contact and droplet precautions, minimizing shared staff, increasing environmental cleaning, and using dedicated equipment. With awareness of the increased community occurrence of RSV, additional measures were taken to monitor adherence to infection prevention and control measures by staff and visitors entering the NICU, including daily symptom screening. Visitation was restricted to block scheduling to monitor the number of individuals in the NICU. Once we obtained the complete conversion of the initial neonate cohort, the additional focus shifted to maintaining the enhanced precautions until all neonate laboratory tests were negative. The NICU was successfully reopened once the remaining 3 positive neonates received no growth on their viral culture. **Conclusions:** The quick and effective response from a multidisciplinary team allowed a successful intervention to mitigate the identified outbreak. This investigation highlights the importance of enhanced infection prevention and control practices during increased community spread. Future efforts focus on educating staff and visitors on appropriate measures to decrease transmission risks.

**Figure:**
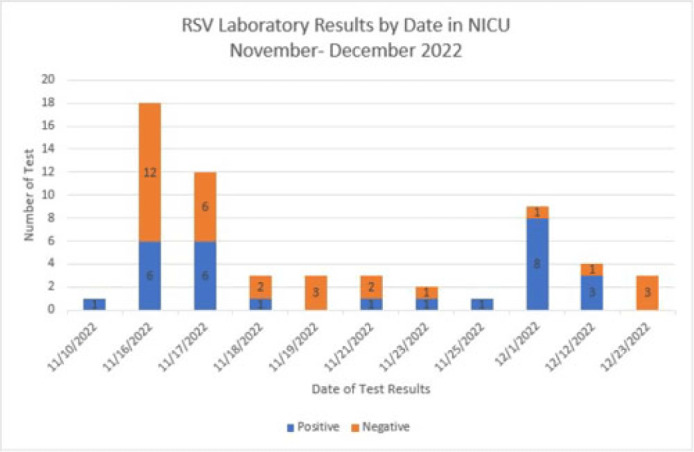

**Disclosures:** None

